# Improving the clinical understanding of hypertrophic cardiomyopathy by combining patient data, machine learning and computer simulations: A case study

**DOI:** 10.1016/j.morpho.2019.09.001

**Published:** 2019-12

**Authors:** A. Lyon, A. Mincholé, A. Bueno-Orovio, B. Rodriguez

**Affiliations:** a Department of Computer Science, University of Oxford, Oxford, United Kingdom; b Cardiovascular Research Institute Maastricht (CARIM), Maastricht University, Maastricht, Netherlands

**Keywords:** Hypertrophic cardiomyopathy, Electrocardiography, e-cardiology, Phenotyping, Computational clustering, Computer modeling, Personalized simulations, Cardiac magnetic resonance imaging, VPH

## Abstract

Most patients with hypertrophic cardiomyopathy (HCM), the most common genetic cardiac disease, remain asymptomatic, but others may suffer from sudden cardiac death. A better identification of those patients at risk, together with a better understanding of the mechanisms leading to arrhythmia, are crucial to target high-risk patients and provide them with appropriate treatment. However, this currently remains a challenge. In this paper, we present a successful example of implementing computational techniques for clinically-relevant applications. By combining electrocardiogram and imaging data, machine learning and high performance computing simulations, we identified four phenotypes in HCM, with differences in arrhythmic risk, and provided two distinct possible mechanisms that may explain the heterogeneity of HCM manifestation. This led to a better HCM patient stratification and understanding of the underlying disease mechanisms, providing a step further towards tailored HCM patient management and treatment.

## Highlights

• In this paper, we present how, by combining electrocardiogram and imaging data, machine learning and high performance computing simulations, we identified four phenotypes in hypertrophic cardiomyopathy (HCM), with differences in arrhythmic risk, and provided two distinct possible mechanisms that may explain the heterogeneity of HCM manifestation.
• This led to a better HCM patient stratification and understanding of the underlying disease mechanisms, providing a step further towards tailored HCM patient management and treatment.

## Résumé

La plupart des patients atteints de cardiomyopathie hypertrophique (CMH), une maladie cardiaque génétique, sont souvent asymptomatiques, mais cette maladie est la cause première de mort subite chez les jeunes. Une meilleure identification des patients à risque, et une meilleure compréhension des mécanismes responsables des arythmies est essentielle pour identifier ces patients à haut risque et leur fournir un traitement approprié. Cependant, cela reste un défi. Dans cet article, nous présentons un exemple réussi de l’implémentation de techniques informatiques pour une application clinique. En associant données d’imagerie et d’électrocardiographie, machine learning et modélisation informatique, nous avons identifié quatre phénotypes parmi la population de CMH, présentant différents niveaux de risque d’arythmie et de mort subite, et avons proposé deux mécanismes possibles pour expliquer l’hétérogénéité observée dans la CMH. Cette étude a donc amelioré la stratification du risque chez les patients atteints de CMH et une meilleure compréhension des mécanismes sous-jacent à la maladie, fournissant une avancée vers une prise en charge individuelle de ces patients et un traitement personnalisé.

## Introduction

Cardiovascular disorders remain a major burden worldwide and are responsible for 30% of deaths in the world. Among them, hypertrophic cardiomyopathy (HCM) is a genetic cardiac disease characterized by the thickening of the left ventricular muscle of the heart, and it is a major cause of sudden cardiac death (SCD), especially among young adults and athletes [1]. Most patients with HCM remain asymptomatic with normal life expectancy, but some of them may die suddenly of cardiac arrest with no previous signs. Getting a better clinical understanding of this heterogeneous clinical course and detect high-risk patients to provide them with appropriate treatment is therefore a challenge and a priority in the management of HCM [2].

HCM hearts suffer from structural changes such as hypertrophy [3], cardiomyocyte disarray [4], fibrosis [5], as well as ion-channel dysfunction [6], which may create an unstable electrical milieu predisposing to arrhythmia. In the past, non-invasive tools to assess the heterogeneity of the HCM population have been developed based on the electrocardiogram (ECG) [7], [8], but they lack specificity to identify the patients at higher risk [9], [10]. In the absence of reliable ECG biomarkers, conventional risk factors (non-sustained ventricular tachycardia, unexplained syncope, family history of SCD, massive left ventricular hypertrophy and abnormal exercise blood pressure response) are also used to evaluate the arrhythmic risk of the patients, and a validated HCM Risk-SCD prediction model has been proposed in 2014 [11], but still shows limitations [12]. Moreover, these clinical observations do not capture the amount of underlying myocardial abnormalities that may lead to arrhythmia, and the pathophysiological mechanisms that may increase HCM risk are still poorly understood.

**Figure 1:**
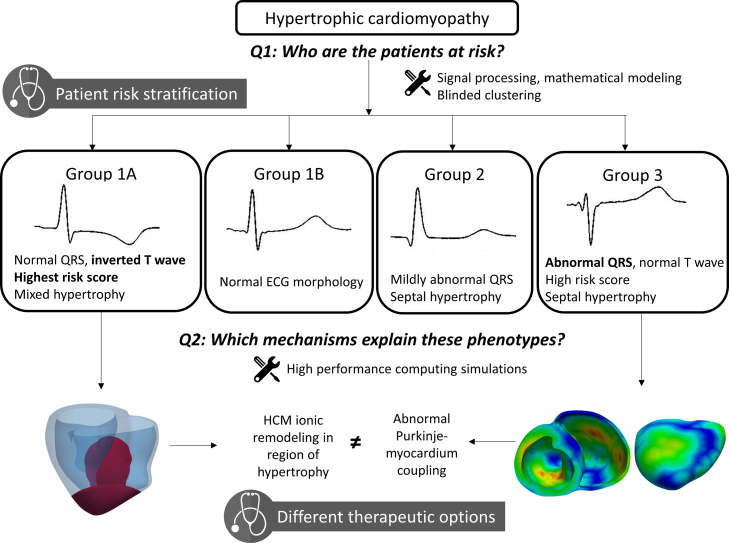
Approach and clinical impact of the case-study. Using signal processing, mathematical modeling and clustering, we identified four different phenotypes in hypertrophic cardiomyopathy based on the ECG. This had a clinical impact by providing improved patient risk stratification. High performance simulations then investigated the potential mechanisms underlying these phenotypes. This provided new options for individual patient management and different therapeutic approaches.

In this translational case study, we present how the use of computational methods such as machine learning and high performance computing simulations helped improve the risk stratification of HCM patients and shed light on the mechanisms underlying the HCM disease. Fig. 1 provides an overall summary of the findings and potential clinical impact of the case study presented in this paper. In a first part, we report how the development of a clustering algorithm based on novel morphological biomarkers derived from the ECG helped identify four distinct phenotypes among the HCM population, which exhibited differences in arrhythmic risk and distribution of left ventricular hypertrophy. In a second part, we show how the use of high performance computing simulations based on clinical cardiac magnetic resonance (CMR) images provided mechanistic understanding of the different ECG phenotypes, and helped to better understand the heterogeneity of HCM. This paper describes how, by developing novel computational technologies for analysis, integration and augmentation of clinical data, we contributed to advancing clinically-relevant insight into HCM and made a step towards individual patient management.

## Methods and results

### ECG-based morphological markers identify four distinct HCM phenotypes that associate with different arrhythmic risk

In the first part of this project, we aimed at developing mathematical modeling and machine learning methods to identify ECG biomarkers that may help improve the understanding and characterization of the heterogeneity of the HCM population [13].

**Figure 2:**
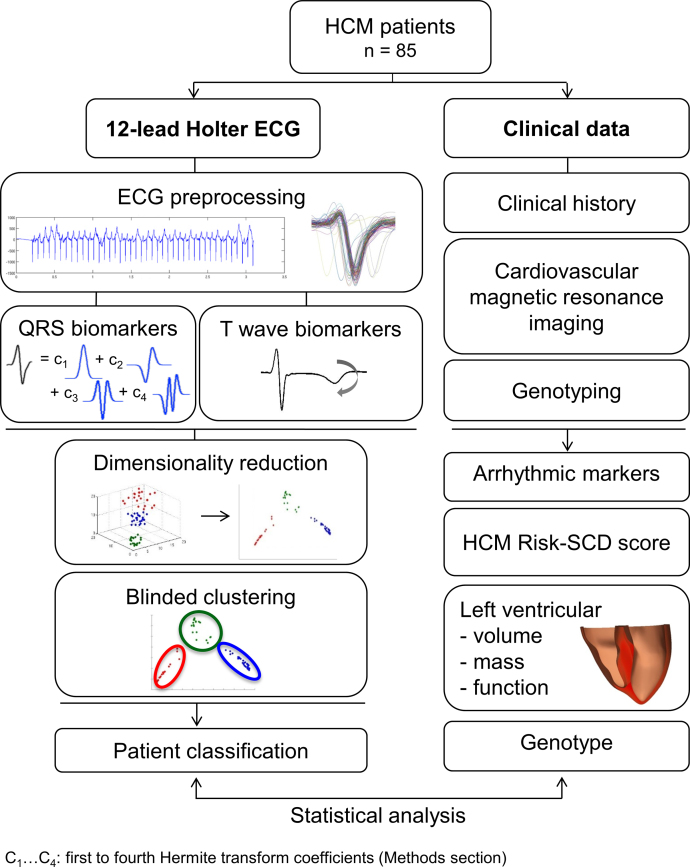
Methodological approach to the identification of four HCM phenotypes (from [13]).

To this end, we analyzed high-fidelity ECG recordings from 85 HCM patients and 38 healthy volunteers recruited as part of a prospective study from the John Radcliffe Hospital in Oxford, UK. 12-lead ECGs, measuring the cardiac electrical activity from twelve different perspectives on the body surface, were recorded for 24 hours using Holter monitors. CMR imaging was also performed for these patients and provided information on the extent and distribution of the hypertrophy. Finally, genetic information, conventional risk factors and family history were obtained as part of routine examination. We then developed signal processing and mathematical modeling tools to compute biomarkers from the different ECG waves in order to characterize their morphology. We focused specifically on the QRS complex, representing the electrical activation of the ventricles, and the T wave, characterizing ventricular relaxation. Standard biomarkers such as amplitude and width of the waves were measured. The QRS shape was also characterized by a combination of Hermite basis, well-established mathematical functions able to provide a compact description of the QRS complex. Four Hermite functions allowed to capture HCM heterogeneity, and each patient was characterized by a vector of morphological QRS and T wave biomarkers. We used an unsupervised feature selection approach combined with a clustering algorithm to investigate and extract subgroups from the HCM population. This was performed blinded to the clinical data. Statistical analysis was finally performed to compare the risk markers between the groups. Fig. 2 summarizes the methodological approach taken.

Based on QRS morphology only, three HCM subgroups were identified. Patients in Group 1 (52% of the population) displayed normal QRS morphology. Group 2 (22% of patients) showed differences in the first three Hermite bases in lead V4 compared to healthy volunteers and Group 1, but no differences in V6. On the ECG, this was observed as a shorter R wave duration and deeper S waves in lead V4 compared to Group 1. Group 3 (26% of patients) exhibited large differences in lead II and V4–V6 compared to the other HCM groups and healthy volunteers, more specifically short R wave duration and amplitude, and longer S wave duration and amplitude. Among these three groups, ECG features were therefore significantly different, but clinical markers and markers of arrhythmic risk were not. This suggested that QRS biomarkers alone were not sufficient for HCM risk stratification.

**Figure 3:**
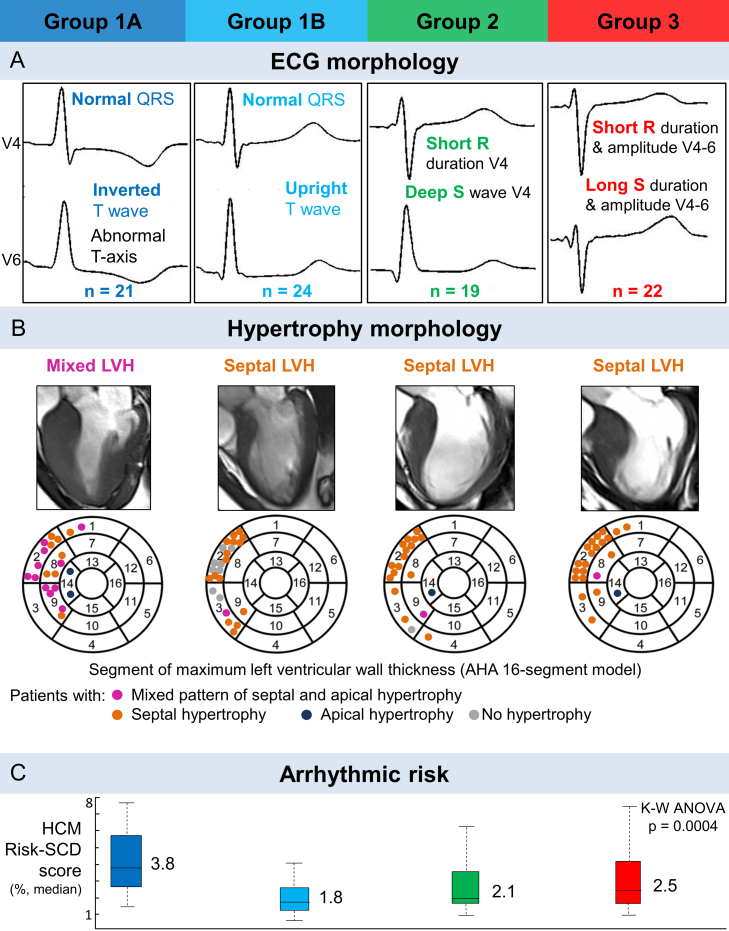
Four phenotypes were identified in HCM, exhibiting differences in ECG morphology (A), distribution of hypertrophy (B) and arrhythmic risk (C) (from [13]).

We then combined both QRS morphology and T wave biomarkers in the clustering algorithm. This led to the identification of four distinct subgroups. Groups 2 and 3 remained the same than the ones identified with QRS features only. However, the addition of the polarity of the T wave as a biomarker separated Group 1 into Group 1A, with inverted T waves in leads V4–V6, and Group 1B, with upright T waves in these leads (Fig. 3, Panel A). Interestingly, Group 1A, with normal QRS morphology but inverted T waves, showed the highest median HCM Risk-SCD score among the four groups (Fig. 3, Panel C). Group 1A also exhibited the most patients with a mixed hypertrophy distribution, with combined septal and apical hypertrophy, while Groups 2 and 3 had mostly septal hypertrophy only. Group 1B patients exhibited little to no hypertrophy (Fig. 3, Panel B).

This study therefore identified four distinct HCM phenotypes based on newly developed ECG morphological biomarkers extracted from high-fidelity recordings. Such phenotypes showed differences in arrhythmic risk scores and distribution of left ventricular hypertrophy. Thus, our study showed the potential of using ECG phenotyping combined with machine learning to dissect the heterogeneity of HCM and help improve individual patient management.

### High performance computer simulations based on CMR images provide different mechanisms for ECG abnormalities in HCM

The previous study identified four subgroups in the HCM population that showed differences in arrhythmic risk and distribution of hypertrophy. Following up on this work, our next objective was to understand the mechanisms behind such a phenotypic heterogeneity, and provide potential explanations for the different subgroups identified [14]. Clinically, improving the mechanistic understanding of HCM may yield a key impact on the individual management of these patients, including potential treatment and tailored therapies.

To this end, we developed a high performance computer simulation framework based on CMR imaging data. We selected representative patients from Group 1A (with normal QRS morphology and inverted T waves, and highest risk score for sudden cardiac death), Group 1B (with normal ECG morphology) and Group 3 (with abnormal QRS, normal T wave, and second highest risk score). From the CMR images of these patients, we computed a personalized volumetric mesh of each patient's heart and torso. The electrical activity across the ventricles was then defined by implementing a cellular computational action potential model [15] at each node of the volumetric mesh, to simulate propagation of the electrical signal. Virtual electrodes were additionally modelled to record the simulated ECG on the virtual patients. With this simulation framework, our aim was to reproduce the ECG abnormalities identified in the different HCM phenotypes of the previous study, and provide potential mechanistic explanations for this ECG heterogeneity using computer simulations. We therefore evaluated the influence of various abnormalities reported in HCM on the ECG morphology, including increased wall thickness, cardiomyocyte fiber disarray, changes in conduction velocity, ionic remodeling or abnormal coupling between the Purkinje fast conduction layer and the myocardium.

**Figure 4:**
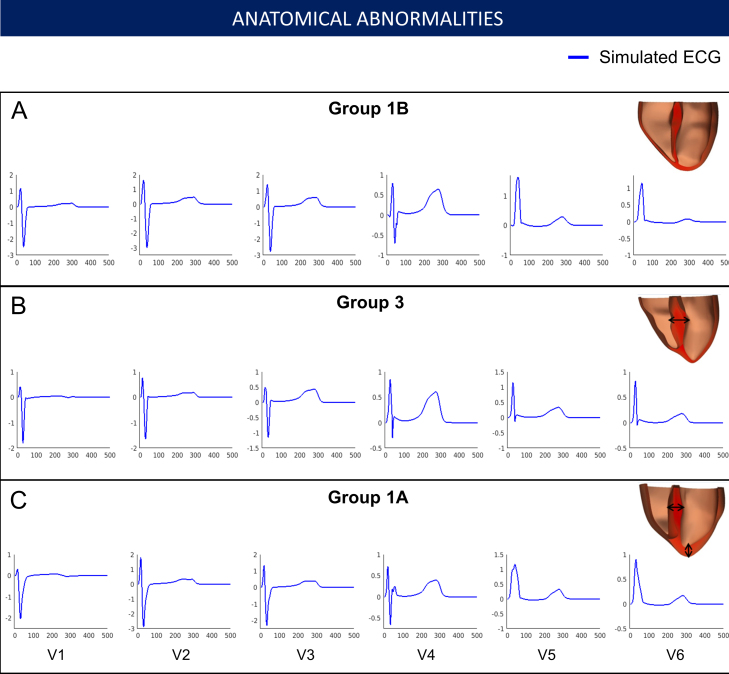
Effect of the anatomy on the ECG. Groups 1B, 3 and 1A exhibit similar ECG morphologies despite differences in extent and distribution of hypertrophy. Anatomy alone does not explain Group 3 and 1A specific abnormalities. Taken from [14].

**Figure 5:**
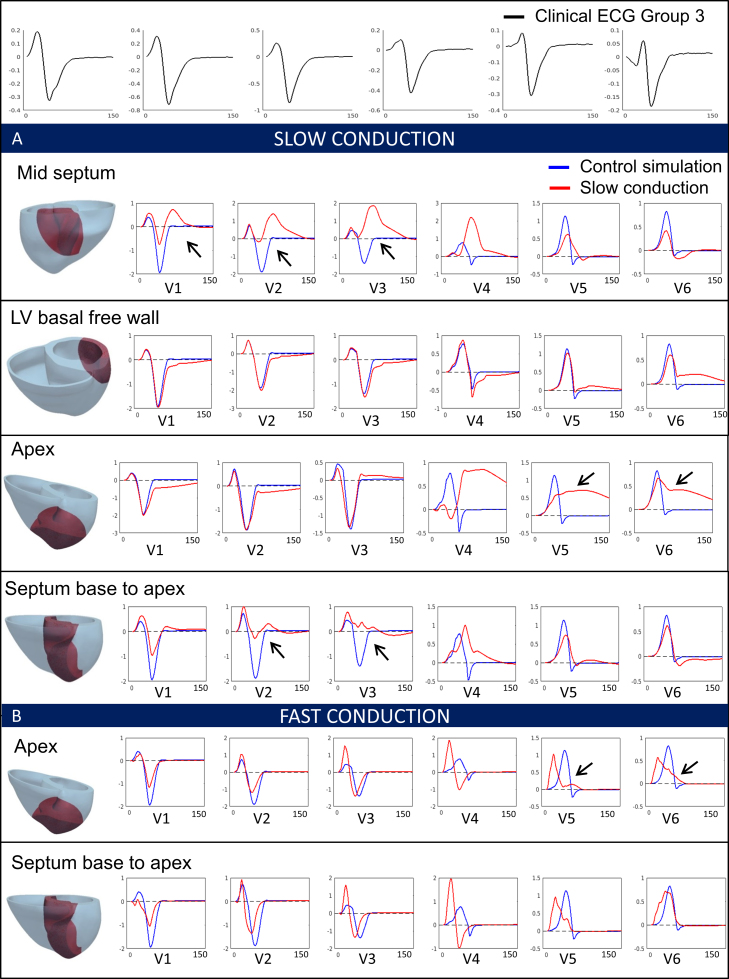
Effect of conduction changes in various regions of the myocardium on the QRS complex (from [14]).

**Figure 6:**
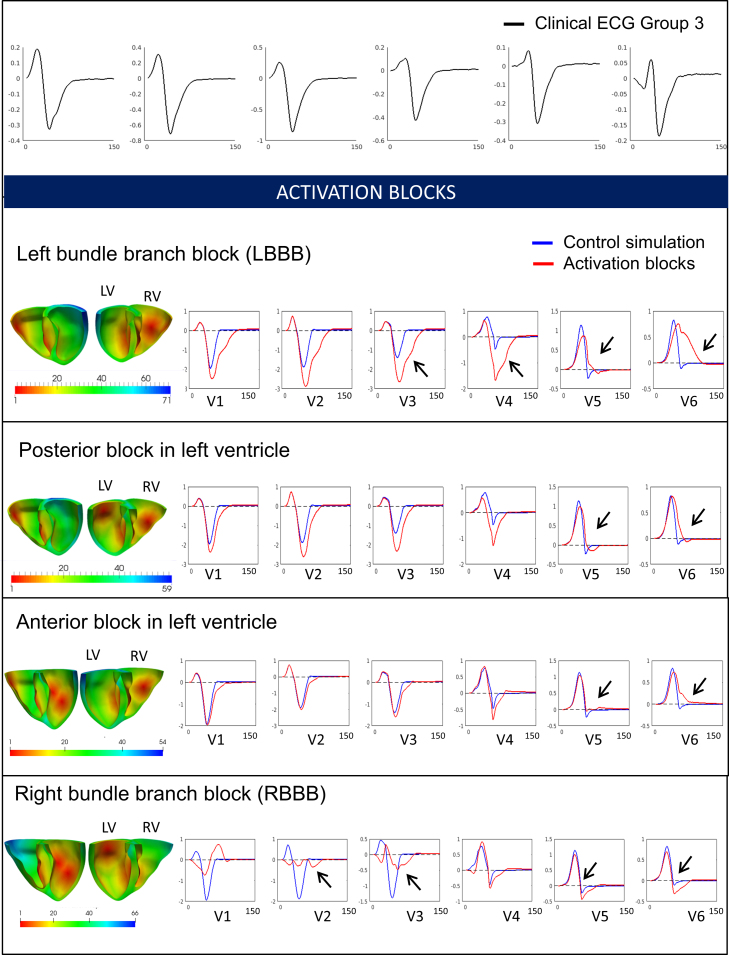
Effect of activation blocks in various regions of the myocardium on the QRS complex (from [14]).

**Figure 7:**
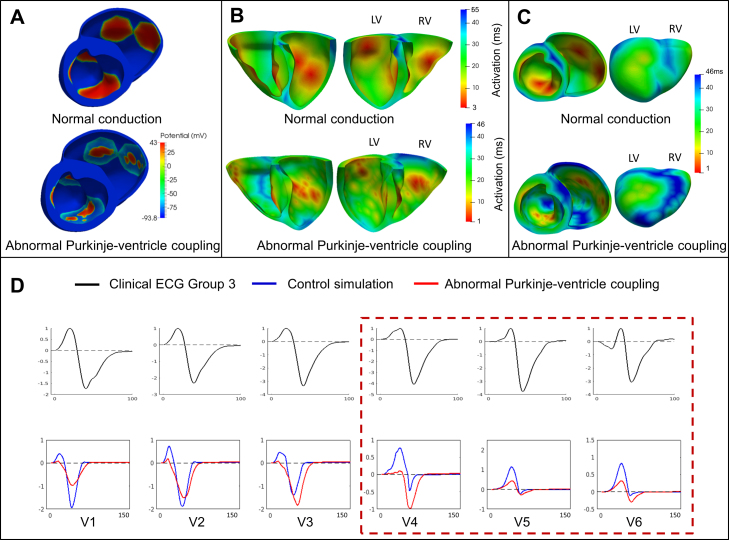
Abnormal Purkinje-myocardium coupling led to a patchy activation (A, B) with areas of late activation (C), and translated into deep S waves in lead V6, explaining Group 3 abnormalities. Taken from [14].

**Figure 8:**
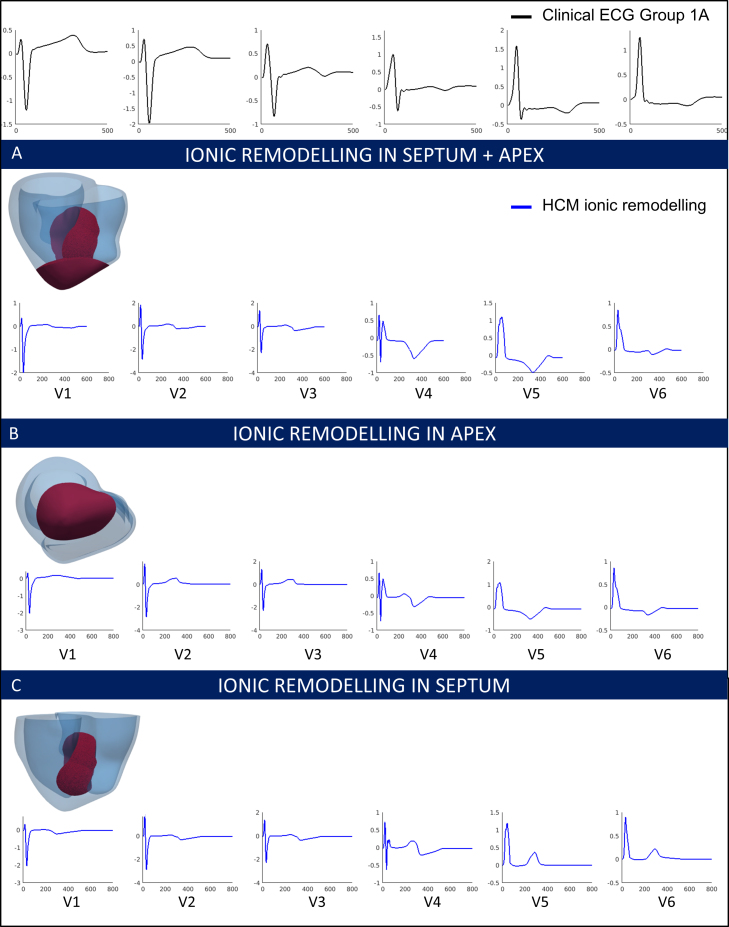
HCM ionic remodeling in various regions of the myocardium. Ionic remodeling in the region of hypertrophy (A) explains the inverted T waves with normal QRS observed in Group 1A patients [14].

These different simulations allowed us to investigate the individual effects of these HCM abnormalities on the ECG and identify those that may be responsible for the HCM phenotypes identified. The distribution of hypertrophy and the anatomy of the patient yielded similar QRS morphology and normal T waves in all cases (Fig. 4). Therefore, increased wall thickness could not explain the QRS and T wave abnormalities observed in Groups 1A and 3. We then focused on the impact of tissue microstructure on the ECG, and evaluated the effect of fiber disarray and altered conduction velocity due to fibrosis or hypertrophy in various regions of the myocardium (such as the septum, or the apex). These led to abnormal QRS complexes, but did not translate into the specific deep S waves in lead V6 that characterized Group 3 (Fig. 5). We then focused on the influence of altering the conduction system by modifying the activation sequence of the ventricles, and the coupling between the Purkinje endocardial layer and the myocardium. Simulating various conduction blocks affected the QRS complexes morphology but did not lead to the abnormalities of Group 3 (Fig. 6). However, modeling an abnormal coupling between the fast endocardial layer and the myocardium was the only way to simulate the deep S waves in leads V4–V6 identified in Group 3 (Fig. 7, Panel D), by creating a patchy electrical activation with areas of late activation (Fig. 7, Panels A to C). Finally, we modelled the HCM ionic remodeling (Fig. 8), including an increase of the late sodium and the L-type calcium currents, a reduction of the potassium currents, and remodeling of the calcium handling subsystem, in hypertrophic areas. This led to a prolonged duration of the action potential in these regions and translated into inversed T waves on the lateral leads of the ECG, explaining the phenotype of Group 1A. These simulations therefore identified two distinct potential mechanisms for the ECG abnormalities associated with an increased risk of SCD in HCM. They also suggested that the HCM ionic remodeling expressed in Group 1A, subgroup with the highest SCD risk score, may play a key role in arrhythmogenesis. The nature of these mechanisms is very different: one is based on conduction abnormalities, while the other involves ionic remodeling. This has implications on the different possible therapies for these groups of patients. Indeed, while Group 1A may benefit from a pharmacological treatment targeting the expression of ion channels, Group 3 may not respond to such therapy.

## Discussion

### Combining computational methods improves the clinical understanding of cardiac diseases

In this paper, we report an example of successful implementation of computational technology for a clinical application in cardiology. The computational methods considered here combine signal processing, mathematical modeling, machine learning and high performance computing, and they contribute to untangle the heterogeneity of HCM and provided more insight in its mechanisms. As illustrated in our case study, the power of these techniques is twofold. Firstly, they are able to make sense of multivariate, complex and heterogeneous datasets and detect differences that might be challenging for the human eye [16]. These techniques make fewer assumptions by selecting discriminatory features from the whole ECG data. With the growing amount of recorded data in clinical settings, the integration of these methods in the clinic may be crucial to aid healthcare decisions and improve patient stratification in large cohorts. Secondly, computational techniques, such as computer modeling, allow the independent assessment of the influence of individual parameters forming a system. This is a key strength compared to standard experimental techniques, for which isolating parameters to study their effect remains a challenge. As a consequence, computational techniques are receiving a growing attention to analyze medical data and address clinical problems [17], [18], [19], and despite that several challenges remain, mostly due to the nature of real-world data (print-out ECGs, incomplete datasets, need for expert consensus), they can help uncover new mechanisms, improve disease knowledge, guide therapies and aid clinical decisions.

### Clinical impact of the work

As mentioned previously in the text, this work showed clinical implications in two ways. First, it provided a new classification of HCM patients based on ECG biomarkers, providing insight in the heterogeneity of the disease. We showed that HCM patients with a primary T wave inversion (and normal QRS) have a greater risk of SCD and arrhythmia, compared to patients with solely QRS abnormalities, highlighting the key role of repolarization in arrhythmogenesis in HCM. We also showed that the location and distribution of hypertrophy was associated to higher SCD risk, rather than the extent of hypertrophy. Secondly, the use of computer simulations provided two distinct mechanisms that may explain the different phenotypes we identified in HCM. Abnormal Purkinje conduction can explain the QRS abnormalities of Group 3, while HCM ionic remodeling in the region of hypertrophy may be responsible for inverted T waves in Group 1A. The fact that Group 1A displayed the highest SCD risk score suggested that ionic remodeling in HCM may play a key role in arrhythmogenesis, while the conduction abnormalities in the Purkinje system may be less proarrhythmic. Finally, this overall better understanding of HCM heterogeneity may have implications in patient management, as personalized treatment for HCM patients could be provided depending on the mechanisms underlying their ECG abnormalities. Indeed, a patient from Group 1A may benefit from a pharmacological treatment improving ion channels function, while this would have no effect on a patient of Group 3.

### Current and potential developments of the work

Developments of the work presented in this paper are currently ongoing. A new database of HCM ECG recordings is being collected and will enable the application of the modeling and clustering technology on a larger dataset. This will allow us to evaluate the ECG criteria presented in the study on a larger cohort with a larger number of cardiovascular end-points and events. This could lead to the definition of a new classification criterion used in the clinic for visual inspection of ECGs in HCM. Moreover, this work generated strong motivation for deeper clinical investigations, such as the use of endocardial or epicardial mapping studies to verify the hypothesis presented here for the mechanisms responsible for the HCM phenotypes. The use of new technologies such as ECG imaging may add even more information to these findings.

## Conclusion

In this paper, we describe the successful integration of computational techniques to improve the clinical understanding of HCM. By combining ECG and CMR imaging data with mathematical modeling, clustering and high performance computing, we identified four distinct HCM phenotypes with differences in arrhythmic risk score, and proposed two different mechanisms that may explain this heterogeneity at the tissue level. This work therefore had impact on personalized HCM patient management and risk stratification, as well as on knowledge and mechanistic understanding of the disease.

## Disclosure of interest

The authors declare that they have no competing interest.
